# Knowledge, attitudes, and practices about HIV and other sexually transmitted infections among High School students in Southern Italy: A cross-sectional survey

**DOI:** 10.1371/journal.pone.0301297

**Published:** 2024-04-19

**Authors:** Francesco Di Gennaro, Francesco Vladimiro Segala, Giacomo Guido, Mariacristina Poliseno, Laura De Santis, Alessandra Belati, Carmen Rita Santoro, Irene Francesca Bottalico, Carmen Pellegrino, Roberta Novara, Luisa Frallonardo, Mariangela Cormio, Michele Camporeale, Sergio Cotugno, Vincenzo Giliberti, Stefano Di Gregorio, Valentina Totaro, Nicola Catucci, Anna De Giosa, Roberta Giusto, Ilaria Viviana Lanera, Gioacchino Angarano, Sergio Lo Caputo, Annalisa Saracino

**Affiliations:** 1 Clinic of Infectious Diseases, Department of Precision and Regenerative Medicine and Ionian Area (DiMePRe-J), University of Bari "Aldo Moro," Bari, Italy; 2 Infection Diseases Unit, Department of Medical and Surgical Sciences, University of Foggia, Foggia, Italy; 3 Associazione CAMA LILA Bari, Bari, Italy; 4 ANLAIDS Puglia Onlus, Bari, Italy; Ruedi Luethy Foundation - Zimbabwe, ZIMBABWE

## Abstract

High School students, recognized as a high-risk group for sexually transmitted infections (STIs), were the focal point of an educational campaign in Southern Italy to share information and good practices about STIs and HIV/AIDS. A baseline survey comprising 76 items was conducted via the REDCap platform to assess students’ initial knowledge, attitudes, and practices (KAP) related to STIs and HIV/AIDS. Sociodemographic variables were also investigated. The association between variables and KAP score was assessed by Kruskal-Wallis’ or Spearman’s test, as appropriate. An ordinal regression model was built to estimate the effect size, reported as odds ratio (OR) with a 95% confidence interval (CI), for achieving higher KAP scores among students features. On a scale of 0 to 29, 1702 participants achieved a median KAP score of 14 points. Higher scores were predominantly reported by students from classical High Schools (OR 3.19, 95% C.I. 1.60–6.33, p<0.001). Additionally, elevated scores were associated with sexually active students (OR 1.48, 95% C.I. 1.12–1.96, p = 0.01), those vaccinated against Human Papilloma Virus (OR 2.47, 95% C.I. 1.89–3.24, p<0.001), those who had used emergency contraception (OR 1.56, 95% C.I. 1.09–2.24, p = 0.02, Table 2) and those obtaining information from TikTok (OR 1.62, 95% C.I. 1.14–2.30, p = 0.01). Conversely, being heterosexual was associated with an overall lower score (OR 0.48, 95% C.I. 0.32–0.73, p<0.001). High School students, often due to early sexual debut, seek information about HIV and STIs independently using social channels. However, the overall level of knowledge, attitudes, and practices remains low. Urgent school-based interventions are needed for this age group.

## Introduction

With over one million daily new infections reported by the World Health Organization (WHO), HIV and sexually transmitted infections (STIs) remain a significant global public health concern, especially for young women and High School students who, undergoing rapid physical growth and substantial psychological changes, face an elevated risk due to sexual experimentation, crucial for the development of individual identity and autonomy [1–4].

The Centers for Disease Control and Prevention (CDC) estimated approximately 2.6 million new STIs in the United States in 2021, with nearly half occurring among individuals aged 15–24; meanwhile, in Italy, the National Epidemiological Observatory reported that the overall number of people with STIs more than doubled between 2004 and 2017, with a particular concern being the prevalence of Chlamydia trachomatis infections in women aged 15–24 years, which was over twice as high as in older women [5, 6].

In this context, the advocacy for comprehensive, school-based, sex education programs addressed to this age group has been linked to postponed initiation and less frequent engagement in sexual activity, heightened usage of condoms, decreased number of sexual partners, and reduced school drop-out rates [7].

However, the preliminary stages of a successful educational campaign involve evaluating the level of awareness among young people regarding HIV and STIs, examining whether they harbor stigmatizing attitudes towards people living with HIV (PLWH), and, most crucially, determining whether they adopt safe sexual behaviors that can effectively prevent the transmission of STIs.

For this reason, this survey aimed to investigate the levels of knowledge, attitudes, and practices (KAP) concerning HIV and other STIs among Italian High School students.

The goal is to use this information as a starting point for implementing targeted interventions and initiatives aimed at improving this special population’s overall awareness and behaviors concerning these health issues.

## Methods

From February 22 to March 28, 2023, an educational and informative campaign on HIV and STIs was conducted for high school students in Southern Italy. The campaign, led by the Unit of Infectious Diseases at Policlinico Hospital in Bari, involved specialists and medical residents in Infectious Diseases, as well as volunteers from patient associations. It comprised twelve sessions held at schools during regular school hours.

In this prospective cross-sectional study, we evaluated the outcomes obtained from the surveys distributed during these sessions, which aimed to gauge the initial levels of students’ knowledge on the subject, understand their attitudes towards discrimination against PLWH, and gather insights into their engagement in safe sexual behaviors. After securing informed consent from both students and parents of minors under 18, data were collected through a self-compiled structured questionnaire on the REDCap platform—a secure web application tailored for building and managing online surveys and databases, supporting both online and offline data capture for research studies and operations [8]. Participants were briefed on the study’s objective, with the assurance of questionnaire anonymity and voluntariness, and three medical doctors from the Infectious Diseases Department of Policlinico of Bari oversaw the questionnaire collection, being available to address any questions in the classroom.

Our working group designed the survey tool, adapting it from a validated questionnaire for assessing brief HIV knowledge [9], with its content validity independently evaluated by all co-authors. A pilot version of the questionnaire was initially emailed to two students from each of the 14 schools to assess face validity and identify potential confounding elements.

The interview consisted of 76 items, encompassing both yes/no and multiple-choice questions. These questions were categorized into four sections: demographic information (7 questions), STI knowledge (43 items), attitudes (9 queries exploring discrimination towards people living with HIV/STIs), and practices related to HIV and STIs (17 questions investigating the prevalence of high-risk sexual behaviors).

All questions were reported in detail and designed to encourage students to open up and maximize their understanding of the questionnaire. It was estimated to take approximately 20 minutes to complete.

The interpretation of the results was entrusted to a score (the Knowledge-Attitude-Prevention -KAP- score) developed through consensus among the co-authors based on existing literature. Responses provided by the students to the 76 questions were condensed into 29 concise items with yes/no answers.

The questionnaire items were organized into three sections, considering the respective dimensions of the categories in the original questionnaire. These sections included 14 items in the Knowledge section, seven in the Attitude, and eight in the Practice section. For each item, a "positive response," indicating good knowledge, attitude, and practices regarding HIV and STIs, was assigned 1 point, regardless of whether it was a "yes" or "no" response. This scoring system allowed for a maximum overall score of 29 points and 14, 7, and 8 points for each subsection of the questionnaire, respectively.

### Statistical analysis

A descriptive analysis was performed to define the distribution of demographic and other variables collected in the KAPsurvey. The Endpoint of the study was the KAP score on sexually transmitted infections (0–29 points).

The distribution of continuous variables was assessed with the Shapiro-Wilk normality test. The association between variables and KAP score was assessed by Kruskal-Wallis’ or Spearman’s test, as appropriate.

An ordinal regression model was implemented as follows. KAP score was considered as the dependent variable. The effect sizes were reported as odds ratio (OR) with 95% confidence interval (CI). The model included a set of clinically relevant candidate predictors and variables that proved significant in the bivariate analysis. Statistical analyses were performed using R Statistical Software (v4.3.1; R Core Team 2021) in R Studio Version 17.

### Ethics statement

The Study was approved by Ethics Committee Azienda Ospedaliero Universitaria Policlinico of Bari, Study n. 7147 on January 12, 2022.

## Results

The educational campaign on HIV and STIs encompassed 14 high schools, including humanities (1 school), science (1 institute), art (2 centers), languages (3 schools), and technical orientation (7 institutes). Among them, 12 schools were situated in the Apulia and 2 in the Abruzzo region. A total of 1,955 students took part in the meetings, the majority (1,631 participants,96%) from Apulia. One thousand seven hundred two students, a median of 16 (Interquartile Range–IQR 16–17) years of age, consented to participate in the survey.

Most respondents were female, cisgender (924 participants, 54.3%), and were attending schools with a technical or professional orientation (876 students, 51.5%).

Table 1 provides a summary of participant details and average questionnaire scores, stratified according to various factors.

**Table 1:** Sociodemographic characteristics and questionnaire scores by participant characteristics.

	Overall (N = 1702)	Stratified KAP score, median (Q1-Q3)	p-value for higher overall KAP score ^1^
**Age**			
Median [Q1, Q3]	16.0 [16.0, 17.0]	-	**<0.001**
Missing	109 (6.4%)	-	
**School type**			
Artistic High School	209 (12.3%)	12(8–15)	**<0.001**
Classical High School	59 (3.5%)	16(13–18)	
Human Sciences High School	104 (6.1%)	12(7–15.75)	
Linguistic High School	290 (17.0%)	13(8–15.25)	
Scientific High School	48 (2.8%)	11(6–13)	
Other	13 (0.8%)	8(0–17)	
Technical or Professional Institute	876 (51.5%)	10(5–14)	
Missing	103 (6.1%)	-	
**School year**			
Median [Q1, Q3]	3.00 [3.00, 4.00]	-	**<0.001**
Missing	121 (7.1%)	-	
**Gender**			
Female	924 (54.3%)	12(7–15)	**<0.001**
Male	605 (35.5%)	10(5–14)	
Non-binary	32 (1.9%)	9(4.75–14)	
Rather not say	33 (1.9%)	12(6–16)	
Missing	108 (6.3%)		
**Sexual orientation**			
Bisexual	156 (9.2%)	12(9–15)	**0.01**
Eterosexual	1365 (80.2%)	11(6–14)	
Lesbian	41 (2.4%)	13(8–16)	
MSM	28 (1.6%)	11.5(0–14)	
Missing	112 (6.6%)	-	
**Region**			
Abruzzo	71 (4.2%)	9(0–13)	0.1
Apulia	1631 (95.8%)	11(5–14)	
**Heard about STIs from parents**			
Yes	690 (40.5%)	12(8–15)	**<0.001**
No	1012 (59.5%)	9(1–14)	
**Heard about STIs at school**			
Yes	618 (36.3%)	12(8–16)	**<0.001**
No	1084 (63.7%)	9(3–14)	
**Heard about STIs from friends**			
Yes	471 (27.7%)	12(7–15)	**<0.001**
No	1231 (72.3%)	10(4–14)	
**Heard about STIs from doctors**			
Yes	439 (25.8%)	13(8–16)	**<0.001**
No	1263 (74.2%)	10(4–14)	
**Heard about STIs from television**			
Yes	893 (52.5%)	11(7–15)	**<0.001**
No	809 (47.5%)	9(0–14)	
**Heard about STIs from Social Media**			
Yes	887 (52.1%)	12(8–15)	**<0.001**
No	815 (47.9%)	8(0–13)	
**Heard about STI on Instagram**			
Yes	688 (40.4%)	13(8–16)	**<0.001**
No	1014 (59.6%)	9(1–13)	
**Heard about STI from TikTok**			
Yes	664 (39.0%)	12(8–16)	**<0.001**
No	1038 (61.0%)	9(1–13)	
**Heard about STI on Twitter**			
Yes	67 (3.9%)	12(8–15)	**0.046**
No	1635 (96.1%)	10(5–14)	
**Heard about STIs from Facebook**			
Yes	88 (5.2%)	12(6.75–15)	0.067
No	1614 (94.8%)	10(5–14)	
**Favorable to Sex Education in Schools**			
Yes	1254 (73.7%)	12(9–15.75)	**<0.001**
No	43 (2.5%)	7(5–10)	
I don’t know	80 (4.7%)	6.5(3–9)	
Missing	325 (19.1%)		
**Being sexually active**			
Yes	617 (36.3%)	14(10–16)	**<0.001**
No	583 (34.3%)	11(8–14)	
Rather not say	88 (5.2%)	8(4–12)	
Missing	414 (24.3%)		
**Intravenous Drug Use**			
Yes	38 (2.2%)	8.5(6.25–12.75)	**<0.001**
No	1176 (69.1%)	12.5(9–16)	
Rather not say	71 (4.2%)	8(3.5–12)	
Missing	417 (24.5%)		
**Shared Syringes (If applicable)**			
Yes	36 (2.1%)	8(5.75–13)	**<0.001**
No	1006 (59.1%)	12(9–16)	
Rather not say	82 (4.8%)	8(5–12)	
Missing	578 (34.0%)		
**Having resorted to emergency contraception**			
Yes	138 (8.1%)	14(10–16.75)	**<0.001**
No	1029 (60.5%)	12(9–15)	
Rather not say	92 (5.4%)	8(4–10)	
Missing	443 (26.0%)		
**HPV Vaccination**			
Yes	768 (45.1%)	13(10–16)	**<0.001**
No	336 (19.7%)	11(7–14)	
Rather not say	167 (9.8%)	10(6–13)	
Missing	431 (25.3%)	-	
**Number of sexual partners this year**			
1	475 (27.9%)	14(10–16)	**<0.001**
2–3	200 (11.8%)	13(9–16)	
4–7	60 (3.5%)	11(8–14.25)	
8+	64 (3.8%)	10(6–13)	
Rather not say	377 (22.2%)	11(8–14)	
Missing	1 (0.9%)	-	

MSM: Males who have Sex with Males; STI: Sexually Transmitted Infections; IV: IV: Intra Venous; HPV: Human Papilloma Virus

^1^Kruskal Wallis or Spearman rank test, as appropriate

Despite their youth, 617 students (36.3%) self-reported sexual activity, with over 30% indicating multiple sexual partners in the past year. Predominant concerns among the surveyed population regarding sexual activity included unwanted pregnancy (70%) and the potential contraction of sexually transmitted infections (57%). Nevertheless, specific inquiries exposed no or infrequent use of barrier contraceptive methods, with 26% and 24% reporting such practices, respectively. Noteworthy is the disclosure from 154 students (10%) having already relied on emergency contraception. Additionally, 38 students admitted intravenous drug usage, with 36 acknowledgingsyringe sharing, constituting 2% of the total population.

Ninety-five percent of the interviewed population stated that they had heard about STIs, primarily HIV infection (89%) and HPV (68%), followed by Monkeypox, Hepatitis B, and Hepatitis C.

Television (52.5%), along with social networks such as Instagram (40.4%) and TikTok (39%,), emerged as the primary information channels for the surveyed population. Only one-third of participants (618, 36%) declared to have heard about STIs at school, although more than 70% declared to be interested in starting courses in sexual education.

Indeed, responses to more in-depth questions revealed that the students had a superficial knowledge of the survey topics.

For instance, one out of every three participants admitted that they didn’t know whether HIV was a rare disease. An even larger group of students, approximately 70%, mentioned that they were unaware of the distinction between HIV and AIDS, the treatment options available for PLWH), and the living conditions and life expectancy of these patients. However, regarding attitudes toward scenarios involving the seropositive status of a friend, teacher, or family member, a substantial majority, approximately 70% of the surveyed student population, exhibited an inclusive and non-discriminatory stance.

About infections such as Chlamydia, Gonorrhea, and Syphilis, a notable lack of awareness was observed. Specifically, 65% of students expressed uncertainty regarding the prevalence of Chlamydia and Gonorrhea infections as rare medical conditions. Additionally, approximately 70% demonstrated unawareness of the symptoms and available treatment options for these STIs.

**Fig 1:**
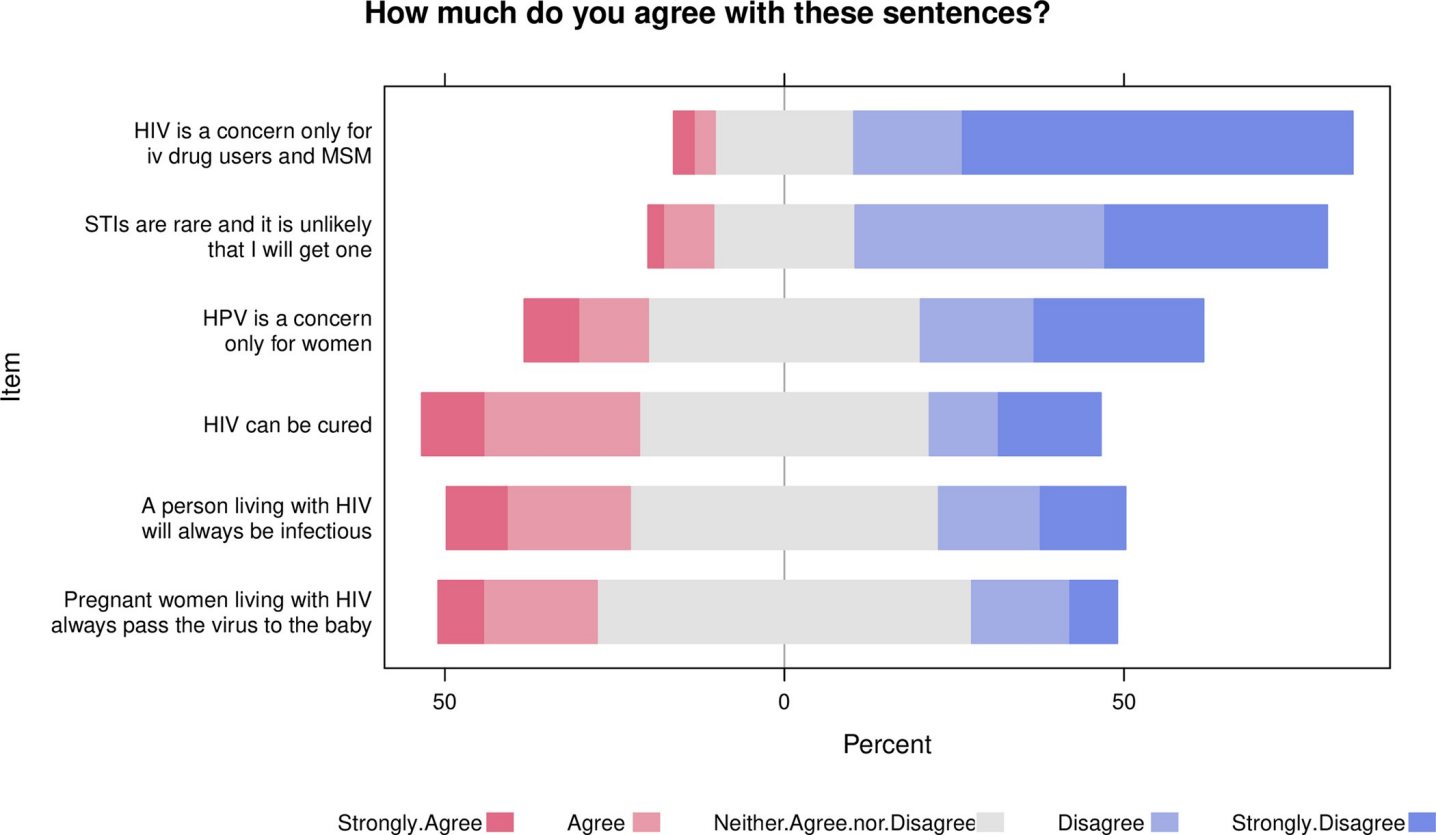
Likert scale chart depicting responses to questions regarding the knowledge of basic concepts regarding HIV and STIs. Likert scale chart depicting responses to questions regarding the knowledge of basic concepts regarding HIV and STIs. Ratings range from strongly disagree (1) to strongly agree (5). Higher scores reflect sufficient knowledge, while lower scores indicate poor understanding of fundamental concepts about STIs and HIV, highlighting the need to spread information about these topics.

The Likert scale chart in **Fig 1** depicts some of the students’ responses to some of the questions asked.

Finally, low adherence to STI screening practices was detected.

Only 46 students (3%) reported ever having undergone an HIV test and only 60 (4%) said having screened for any STI. Conversely, Human Papilloma Virus (HPV) vaccination appeared pretty widespread among the student population: 768 students (45%) stated that they were vaccinated against HPV.

Overall, on a scale from 0 to 29, participants achieved a median KAP score of 14 points. Multivariate ordinal regression assessing predictors of higher KAP score are reported in Table 2.

**Table 2:** Multivariate ordinal regression assessing predictors of higher KAP score.

Variables	aOR	Low C.I.	High C.I.	p-value
**Gender (reference: females)**				
Male*	1.15	0.89	1.48	0.28
Nonbinary gender*	0.45	0.16	1.31	0.14
**Sexual identity**				
Heterosexual	0.48	0.32	0.73	**<0.001**
Lesbian	0.63	0.30	1.31	0.21
MSM	1.34	0.51	3.49	0.55
**School type**				
Classical High School	3.19	1.60	6.33	**<0.001**
Human Sciences High School	0.53	0.29	0.98	0.04
Linguistic High School	1.04	0.69	1.56	0.86
Scientific High School	1.44	0.66	3.11	0.36
Technical or Professional Institute	0.73	0.51	1.04	0.08
Other	1.95	0.26	18.38	0.53
**I heard about STI from**				
Parents	1.15	0.91	1.44	0.24
School Teachers	1.24	0.98	1.57	0.08
Friends	0.98	0.76	1.26	0.87
Doctors	1.22	0.94	1.57	0.13
Television	0.95	0.75	1.19	0.63
Social Networks	1.05	0.69	1.60	0.82
Instagram	1.05	0.73	1.50	0.80
TikTok	1.62	1.14	2.30	**0.01**
Twitter	0.87	0.50	1.51	0.61
Facebook	1.25	0.78	2.02	0.35
**Being sexually active**	1.48	1.12	1.96	**0.01**
**Having experienced IV drug use**	1.35	0.62	2.94	0.46
**Having resorted to emergency contraception**	1.56	1.09	2.24	**0.02**
**Being vaccinated against HPV**	2.47	1.89	3.24	**<0.001**
**Number of sexual partners during last year** ^**&**^				
2–3	1.02	0.75	1.40	0.88
4–7	0.76	0.45	1.28	0.30
8+	0.79	0.46	1.35	0.38

aO.R.: adjusted Odds Ratio; C.I.: Confidence Interval; MSM: Males who have Sex with Males; STI: Sexually Transmitted Infections; IV: Intra Venous; HPV: Human Papilloma Virus

* Reference: female sex

Higher scores were predominantly reported by students from classical High Schools (OR 3.19, 95% C.I. 1.60–6.33, p<0.001). Additionally, elevated scores were associated with sexually active students (OR 1.48, 95% C.I. 1.12–1.96, p = 0.01), those vaccinated against HPV (OR 2.47, 95% C.I. 1.89–3.24, p<0.001), and those who had used emergency contraception (OR 1.56, 95% C.I. 1.09–2.24, p = 0.02, Table 2). Notably, obtaining information from TikTok was a strong predictor of above-average KAP scores (OR 1.62, 95% C.I. 1.14–2.30, p = 0.01), indicating a higher likelihood of achieving superior KAP scores. Conversely, being heterosexual was associated with an overall lower score (OR 0.48, 95% C.I. 0.32–0.73, p<0.001).

## Discussion

Recognizing the increased vulnerability of adolescents to sexually transmitted infections, primarily associated with the initiation of sexual activity during this period, the Italian Ministry of Education has recently acknowledged the significance of integrating sexual and emotional education courses into school curricula [10].

However, to date, there is limited available data regarding the baseline level of knowledge about STIs and HIV among individuals in school age [11, 12].

Our survey, conducted purposely in the context of an informative campaign on this topic, stands as the first of its kind in South Italy.

In this region, given the socioeconomic disparities existing in the Italian country [13], we hypothesized lower levels of awareness and less favorable practices concerning these health issues.

From our experience, we could drawsome relevant observations.

Firstly, we identified a relatively low overall level of general knowledge and prevention of STIs and HIV infection, underscoring the urgent need for an educational intervention to address these gaps. This intervention, primarily designed as a scientific outreach campaigns to be conducted in the school environment, appears even more imperative in light of some information provided by the students, particularly regarding their young age at the onset of sexual activity.

Early sexual debut is reported in 21.7% of Italian High School students, especially among those from a higher socioeconomic background [14, 15]. Evidence suggests that the primary contributing factor to the elevated risk of STIs, unintended pregnancies, and the need for elective abortions among individuals in this age group is the lack of comprehensive sexual education and insufficient awareness on these topics [16].

The survey responses revealed that participants who were already sexually active actively sought information on topics such as STIs, HIV, contraceptive methods, and active prophylaxis. However, the selected information channels for their knowledge acquisition were television and social media. This likely contributed to the level of understanding of the issue, which, although decent, was superficial and imprecise.

These findings prompt reflection on the limited involvement of Italian educational institutions in these outreach activities, a challenge that may be attributed to cultural barriers within the Italian social and cultural context. Despite their inclusion in the curriculum, subjects such as sexually transmitted diseases, HIV, and abortion appear to be effectively excluded from biology or science curricula. This decision is often associated with cultural resistance, resulting in opposition from teachers and parents.

The inadequate institutional engagement on the topic of STIs and HIV becomes evident when examining differences in responses based on sexual orientation. Our survey demonstrates that adolescents identifying as heterosexual were more prone to achieving lower KAP scores compared to their LGBTQ+ peers, indicating a potential discrepancy in sexual health knowledge. This implies that non-binary and LGBTQ+ adolescents may actively pursue sexual health education due to personal experiences of discrimination or stigma, heightened sensitivity to health issues, and improved access to LGBTQ+ resources and support networks. Consequently, they engage more frequently with healthcare professionals, seeking comprehensive information and support [17].

A positive aspect gleaned from our high school experience was the notable adherence to vaccination campaigns against STIs, particularly HPV.

Additionally, participants vaccinated against HPV exhibited higher levels of knowledge, attitudes, and practices, as reflected in their questionnaire scores. In Italy, HPV vaccination is highly recommended and provided free of charge to individuals aged 11 and above [18]. The improved knowledge in this group of students may be attributed to the counseling provided during HPV vaccinations, potentially enhancing their understanding of STI transmission.

On the contrary, responses provided by the population in the section concerning sexual practices and STI prevention have painted a more disheartening picture.

It indicates that in daily life, very few students, despite having a minimal level of knowledge regarding these issues, implement safe sexual behaviors to avoid infection. Condom usage is minimal or nonexistent, many students had already resorted to emergency contraceptive measures, and several reported engaging with multiple partners within the past year. The observed phenomenon can be attributed to several factors, such as a lack of perceived risk, social and peer pressures, deficient relational skills, limited access to sexual health services, inadequate understanding of long-term consequences, emotional impulsivity, and a failure to appreciate STIs’ gravity fully.

Finally, a positive aspect of our study emerged from the questionnaire responses, indicating that students generally maintain an inclusive and non-discriminatory attitude towards individuals living with HIV, despite their limited knowledge about many specific aspects of the condition and a lack of everyday preventive strategies. This observation suggests a potential separation between their attitudes and practical understanding of HIV, prompting further investigation into the underlying factors influencing this divergence.

It is important to note that this study has certain limitations.

Firstly, the questionnaire was exclusively administered to High School students from Southern Italy, and specifically from the Abruzzo and Apulia regions, limiting the generalizability of findings to all Italian students. This territorial focus has been noted in previous Italian surveys, which similarly faced geographical constraints and reported inadequate knowledge [10–12].

Secondly, the questionnaire was voluntary, making it difficult to assess response accuracy. Some participants chose not to answer specific questions, potentially introducing bias.

Nevertheless, our research boasts notable strengths, such as the high number of participants and the comprehensive set of detailed questions formulated. These features facilitated the collection of a wealth of data and provided a solid foundation for the analysis and interpretation of the findings.

## Conclusions

According to the insights provided by this survey, High School students in Southern Italy demonstrate an overall low level of general knowledge regarding STIs primarily acquired through social media. Additionally, despite an early onset of sexual activity being highlighted, only a few adhere to safe sex practices in their daily lives. These findings underscore the crucial role of institutions and families in educating young individuals on these matters, emphasizing the urgent need for enhanced efforts in overcoming prevailing social and cultural barriers that have significantly constrained such education in our country.

## References

[1] The World Health Organization.—Sexually transmitted infections (STIs). Available from: https://www.who.int/news-room/fact-sheets/detail/sexually-transmitted-infections-(stis) (Accessed 13 Mach 2024)

[2] Istituto Superiore di Sanità –Infezioni sessualmente trasmesse–Gruppi a rischio: adolescenti. Available from: https://www.epicentro.iss.it/ist/adolescenti (Accessed 13 Mach 2024)

[3] The World Health Organization—Adolescent health. Available from: https://www.who.int/health-topics/adolescent-health (Accessed 13 Mach 2024)

[4] Brunelli L, Bravo G, Romanese F, Righini M, Lesa L, De Odorico A, et al. Sexual and reproductive health-related knowledge, attitudes and support network of Italian High School students. Public health in practice (Oxford, England), 3, 100253. 2022. doi: 10.1016/j.puhip.2022.100253 36101775 PMC9461229

[5] Center for Disease Control—2018 STD Surveillance Report. Published September 13th, 2021. Available from: https://www.cdc.gov/nchhstp/newsroom/2019/2018-STD-surveillance-report.html (Accessed 13 Mach 2024)

[6] Trani F, Gnisci F, Nobile CGA, Angelillo IF. High School students and sexually transmitted infections: knowledge and behavior in Italy. Journal of Paediatrics and Child Health 2005;41(5–6):260–264. doi: 10.1111/j.1440-1754.2005.00607.x 15953325

[7] Kirby D. The impact of schools and school programs upon adolescent sexual behavior. Journal of Sex Research. 2002;39(1):27–33. doi: 10.1080/00224490209552116 12476253

[8] REDCap Research Electronic Data Capture Available from: https://projectredcap.org, last accessed September 21st, 2023 (Accessed 13 Mach 2024)

[9] Carey MP, Schroder KEE. Development and Psychometric Evaluation of the Brief HIV Knowledge Questionnaire. AIDS Education Prevention 2002;14(2):172–182. doi: 10.1521/aeap.14.2.172.23902 12000234 PMC2423729

[10] Ministero dell’Università e della Ricerca (MIUR) Circolare n. 1211 del 4 maggio 2022, Available from: https://www.miur.gov.it/-/circolare-n-1211-del-4-maggio-2022 (Accessed 13 Mach 2024)

[11] Beltrami C, Manfredi R, D’Antuono A, Chiodo F, Varotti C. Sexually-transmitted infections in High School students and young adults in a large city of Northern Italy: a nine-year prospective survey. New Microbiology 2003;26(3):233–241. 12901418

[12] Drago F, Ciccarese G, Zangrillo F, Gasparini G, Cogorno L, Riva S, et al. A Survey of Current Knowledge on Sexually Transmitted Diseases and Sexual Behaviour in Italian High School students. International journal of environmental research and public health. 2016. 13(4), 422. 10.3390/ijerph13040422 27089354 PMC4847084

[13] Istituto Nazionale di Statistica (ISTAT) Rapporto sul territorio 2020, Available from: Rapportoterritorio2020.pdf (istat.it) (Accessed 13 Mach 2024)

[14] Borraccino A, Lo Moro G, Dalmasso P, Nardone P, Donati S, Berchialla P & the 2018 HBSC-Italia Group. Sexual behavior in 15-year-old adolescents: insights into the role of family, peer, teacher, and classmate support. Annali dell’Istituto superiore di sanita. 2020. 56(4), 522–530. 10.4415/ANN_20_04_17 33346181

[15] Guetto R, Vignoli D, Lachi A. Higher parental socioeconomic status accelerates sexual debut: Evidence from university students in Italy. Advances in Life Course Research. 2022;51:100461. doi: 10.1016/j.alcr.2022.100461 36652315

[16] Alexander CS, Guyer B. Adolescent Pregnancy: Occurrence and Consequences. Pediatric Annals. 1993;22(2):85–88. doi: 10.3928/0090-4481-19930201-05 8493058

[17] Gowen LK., Winges-Yanez N. Lesbian, gay, bisexual, transgender, queer, and questioning youths’ perspectives of inclusive school-based sexuality education. Journal of sex research. 2014. 51(7), 788–800. 10.1080/00224499.2013806648 (Accessed 13 Mach 2024) 24003908

[18] Ministero della Salute–Campagne Vaccinazioni. Available from: https://www.salute.gov.it/portale/vaccinazioni/dettaglioCampagneVaccinazioni.jsp?lingua=italiano&menu=campagne&p=dacampagne&id=167 (Accessed 13 Mach 2024)

